# Simulation study for the energy and position reconstruction performances of the beam monitoring system of Carbon Ion Radiation Therapy using GEANT4

**DOI:** 10.1371/journal.pone.0313862

**Published:** 2025-02-04

**Authors:** Yun Eo, Na Hye Kwon, Joonsuk Bae, Byunggu Cheon, Guk Cho, Suyong Choi, Hyunsuk Do, Seungkyu Ha, Changgi Huh, Kyuyeong Hwang, Haeun Jang, Seoyun Jang, Yoonjun Jang, Jinryong Jeong, Beomkyu Kim, Bobae Kim, Dongwoon Kim, Sungwon Kim, Sanghyun Ko, Hyupwoo Lee, Hyungjun Lee, Jason Lee, Junghyun Lee, Sehwook Lee, Woochan Lee, Yunjae Lee, Sanghoon Lim, Hyesung Park, Jaehyeok Ryu, Jin Sung Kim, Min Sang Ryu, Hwidong Yoo, Dong Wook Kim, Minsuk Kim

**Affiliations:** 1 Department of Physics, Yonsei University, Seoul, South Korea; 2 Department of Radiation Oncology, Yonsei Cancer Center, Heavy Ion Therapy Research Institute, Yonsei University College of Medicine, Seoul, South Korea; 3 Department of Physics, Sungkyunkwan University, Seoul, South Korea; 4 Department of Physics, Hanyang University, Seoul, South Korea; 5 Department of Physics, Korea University, Seoul, South Korea; 6 Department of Physics, Kyungpook National University, Daegu, South Korea; 7 Department of Mathematics and Physics, Gangneung-Wonju National University, Gangneung, South Korea; 8 Department of Physics, Seoul National University, Seoul, South Korea; 9 Department of Physics, University of Seoul, Seoul, South Korea; 10 Department of Physics, Pusan National University, Busan, South Korea; 11 Center for High Energy Physics, Kyungpook National University, Daegu, South Korea; Ural Federal University named after the first President of Russia B N Yeltsin Institute of Physics and Technology: Ural’skij federal’nyj universitet imeni pervogo Prezidenta Rossii B N El’cina Fiziko-tehnologiceskij institut, RUSSIAN FEDERATION

## Abstract

Carbon Ion Radiation Therapy is operated in several countries because of its advantage to have high dose concentration and/or high linear energy transfer (LET). To estimate the beam performance of Carbon Ion Radiation Therapy, we target the 1% energy and 1 *mm*^2^ position resolutions of the beam monitoring system. The beam monitoring system consists of a scintillation crystal and fiber hodoscope. The scintillation crystal is 20 × 20 × 120*mm*^3^ and its candidates are LYSO, CsI and BGO. The fiber hodoscope is composed of 1 *mm* thickness scintillation fibers and the fibers are arranged vertically for 2D reconstruction. With GEANT4 simulation, we verify the performance of our beam monitoring system. The energy response of the LYSO and BGO scintillators is linear within ± 2%. The energy resolution of each crystal candidate achieves the goal; LYSO (0.061%), CsI (0.20%) and BGO (0.10%). The position is reconstructed via fiber hodoscope within 5% uncertainty.

## Introduction

Particle radiotherapy, which employs accelerated carbon ions or protons, has been a groundbreaking clinical technique since the 1950s. The concept of using proton beams for medical purposes was first proposed by Robert R. Wilson in 1946 [1], and the first heavy ion accelerator was developed at the National Institute of Radiological Sciences (NIRS) in Japan [2, 3]. The practice of Carbon Ion Radiotherapy (CIRT) commenced with initial treatments at the Heavy Ion Medical Accelerator in Chiba (HIMAC) in 1994 [2]. Since then, CIRT has evolved into a highly effective form of cancer treatment. Several facilities worldwide have embraced this technology, leading to significant advancements not only in treatment techniques but also in associated equipment [4, 5].

CIRT offers substantial benefits over conventional photon therapy by precisely targeting tumors while minimizing collateral damage to surrounding healthy tissues and organs. Also, the advantage of the CIRT is proved in clinical by several studies [6–10], providing good local control and survival advantage with tolerable adverse effect for rectal cancer, recurrent glioblastoma, hepatocellular carcinoma, prostate cancer and so on. While the evolution of CIRT is marked by continuous improvement in treatment delivery and planning systems, its core principle remains unchanged—exploiting the unique physical and biological properties of carbon ions for precise and efficient tumor control. With its ‘Bragg peak’—a unique energy deposition at a specific depth—and ‘higher relative biological effectiveness (RBE)’, CIRT proves particularly effective for treating deep-seated or complex tumors that are challenging for traditional photon-based therapies [11, 12]. While CIRT represents a significant advancement in radiotherapy (RT), its success heavily relies on precise position and energy measurements of the beam—factors that directly impact treatment quality and patient safety. Misalignment or inaccuracies in energy control could lead to under-treatment of the tumor or unnecessary exposure of healthy tissues to high-energy particles.

This critical importance of Quality Assurance (QA) is underscored by reports such as the AAPM Task Group 224 (TG-224) report published in 2019 [13], which provides guidelines for comprehensive QA procedures in proton therapy. The TG-224 report emphasizes that accurate beam position and energy measurement are key components of an effective QA program, influencing every aspect from treatment planning to delivery. Inaccurate beam positioning can lead to off-target radiation delivery, potentially harming healthy tissues while missing tumor cells [14, 15]. Similarly to CIRT, inaccuracies in beam energy can affect depth-dose distribution, leading to under-dosing or over-dosing issues which compromise treatment effectiveness and patient safety. Therefore, advanced technologies for real-time monitoring and accurate energy measurement are indispensable to ensure precise delivery of CIRT.

In the RT process, QA is critical in verifying that every aspect of the treatment process meets predefined standards. However, despite advancements in CIRT technology, current detectors used for QA have limitations. For instance, the limited spatial resolution of 2-Dimensional ionization chamber array (MatriXX Resolution™, IBA, Schwarzenbruck, Germany) can affect Bragg peak positioning and subsequently impact treatment outcomes. Moreover, existing detectors often struggle with accurate energy measurement at higher particle energies—a challenge that potentially compromises therapy effectiveness. These challenges highlight an urgent need for advanced detector technologies offering improved resolution, real-time monitoring capabilities, accurate high-energy measurements and practicality in busy clinical settings.

According to TG-224, the recommended tolerance range for beam energy is ± 1 *mm*, with spot position tolerances of ± 2 *mm* (absolute) and ± 1 *mm* (relative) to maintain quality assurance in proton therapy [13]. Taku Nakaji et al. demonstrated good agreement between physical and clinical dose calculations using treatment planning systems and Monte Carlo simulations, within 1.0% at the center of spread-out Bragg peaks [16]. Additionally, Eike Rietzel et al. highlighted that accuracy in range calibration of 1% corresponds to approximately a 1 *mm* range control for carbon ions at a water-equivalent depth of 10 *cm*, which is typical for treatment depths in head and neck tumors [17].

Achieving high-position resolution is equally crucial. F. S. Matar et al. utilized solid-state detectors to achieve high spatial resolutions of 0.784 *mm* and 0.2 *mm* in 2020 [18]. Oliveira, A.M. et al. in 2024, developed a detector using GEM-TFT technology that achieved a full width at half maximum (FWHM) below 0.50 ± 0.05 *mm*, corresponding to a resolution of 1 *lp*/*mm*[19]. These recent technologies highlight the potential for meeting rigorous standards for both energy and position resolution, enhancing the quality of radiation treatments.

In response to this need, our research focuses on developing advanced detector system with energy resolution 1% and position resolution 1 *mm*^2^ using scintillation crystals. Crystals detect incoming particles with high precision—a critical factor for accurately targeting tumors while protecting surrounding healthy tissues. Also, each of crystals have advantages as follows: *Lu*_2(1−*x*)_*Y*_2*x*_*SiO*_5_ (LYSO, high light output/fast response) [20, 21], *Bi*_4_*Ge*_3_*O*_12_ (BGO, excellent gamma-ray stopping power) [22], and CsI (moderate light yield/robustness/affordability) [23]. Prior to developing the detector, this study utilizes GEANT4 simulations aiming at evaluating the accuracy and precision as scintillation crystals used for position and energy measurement within QA procedures integral to carbon ion therapy.

## Set up

To achieve the goal of more accurate beam monitoring system, a simulation is performed with various parameters in order to determine the material and configuration of this monitoring system. We discuss the set-ups of the simulation and geometry in this section.

### Simulation set up

The GEANT4 simulation package is used in this study [24, 25]. Since CIRT operates carbon beams in a few hundred MeV ranges, precise description of the interaction between carbons and materials is extremely important. Therefore we choose this program widely utilized in medical and particle physics. The GEANT4 is based on C++ library and we use the version of 10.5.1 [26]. All default parameters are used in this GEANT4 version, and the recommendations of medical physics are also implemented. We use the FTFP_BERT physics list [27], which is suggested for the lowest error among the lists in reference. The comparison with a QGSP_BIC [27], popularly recommended in ion therapy, will be discussed in the result section.

We use the range of carbon beams from 100 to 400 MeV/u in this simulation study. This is because the CIRT at Severance hospital [28] in South Korea accelerates carbons up to a range of 55.6 to 430 MeV/u. Additionally, for cross-checking in the simulation, the performance of the proton beams are also tested from 50 to 200 MeV, which are widely used at ion therapy facilities in the world.

### Geometry set up

A scintillation crystal and a fiber hodoscope are used in order to meet our goal of energy and position resolutions. Three types of scintillation crystals are considered: LYSO, CsI, and BGO. LYSO has high light yield therefore it provides high precision of energy reconstruction. On the other hand, the cost of LYSO [31, 32] is extremely high compared to other scintillation crystals. CsI [23, 33], which has an advantage in the cost, is tested as an alternative candidate despite its low light yield. In addition, BGO [34] is studied as a moderate option of the cost and light yield. The specifications of all three crystal candidates are shown in Table 1.

**Table 1:** The detail scintillation crystal specification is in this table. Light yield and decay time are important parameters of scintillation effect [29]. The photons generated from scintillation effect are affected by refractive index, attenuation length and the density of material [29, 30].

	LYSO (*Lu*_2(1−*x*)_*Y*_2*x*_*SiO*_5_)	CsI	BGO (*Bi*_4_*Ge*_3_*O*_12_)
Density (g/*cm*^3^)	7.25	4.51	7.13
Light yield (photons/MeV)	33200	1900	8000
Decay time (ns)	36	15.7	300
Refractive index	1.82	1.95	2.15
Attenuation length (cm)	42	40	-

**Fig 1:**
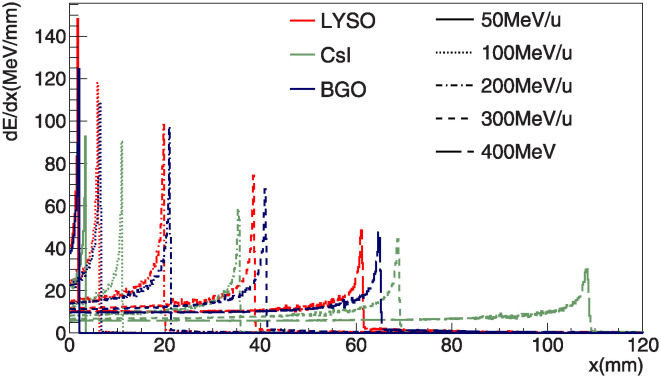
The Longitudinal energy deposit distribution in each crystal is shown at various incident energies ranging from 50 to 400 MeV/u as a function of depth in *mm*. The Bragg peak is shown in each energy of carbon beam. The red, green and blue lines denote LYSO, CsI and BGO respectively.

**Fig 2:**
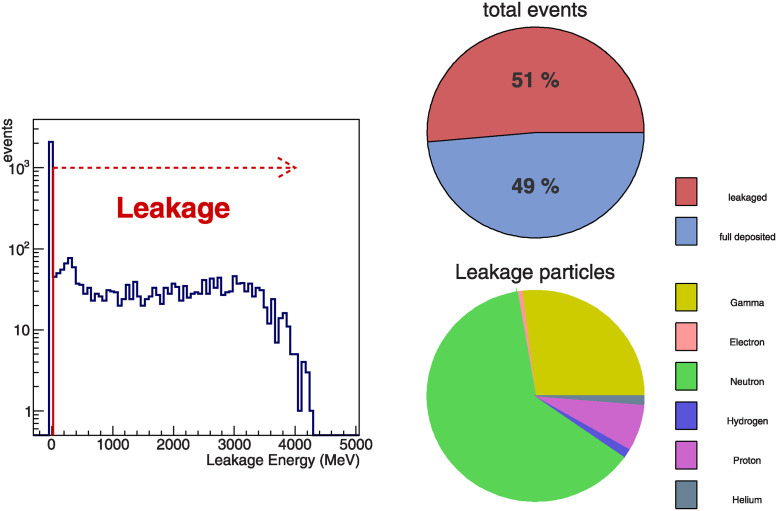
The left plot is distribution of leakage energy from events simulated with 400 MeV/u carbon shot on LYSO crystal. The diagram on the top right indicates the ratio of the number of events between fully deposited and leaked energy. The diagram on the bottom right shows the composition of leakage events, with the dominant leakage originating from neutrons.

For determination of crystal dimension, we check the distribution of deposit energy in the each crystal. Fig 1 shows longitudinal distribution of the absorbed energy. For the specified incident energies used in this study, we observe Bragg peaks for all three crystal candidates within the 120 mm range which is the length of the crystal in our monitoring system. The deposited energy distributions in the transverse direction are over 97% of total deposit energy in crystal within ± 5 *mm* and no significant difference is observed among crystal candidates. However, as shown in Fig 2, half of the total events exhibit leakage, and the dominant source of this leakage is neutrons. Despite the presence of the leakage, half of the events included in the full peak are expected to achieve sufficient energy performance. Finally, we determine the size of the scintillation crystal to be 20 × 20 × 120*mm*^3^.

**Fig 3:**
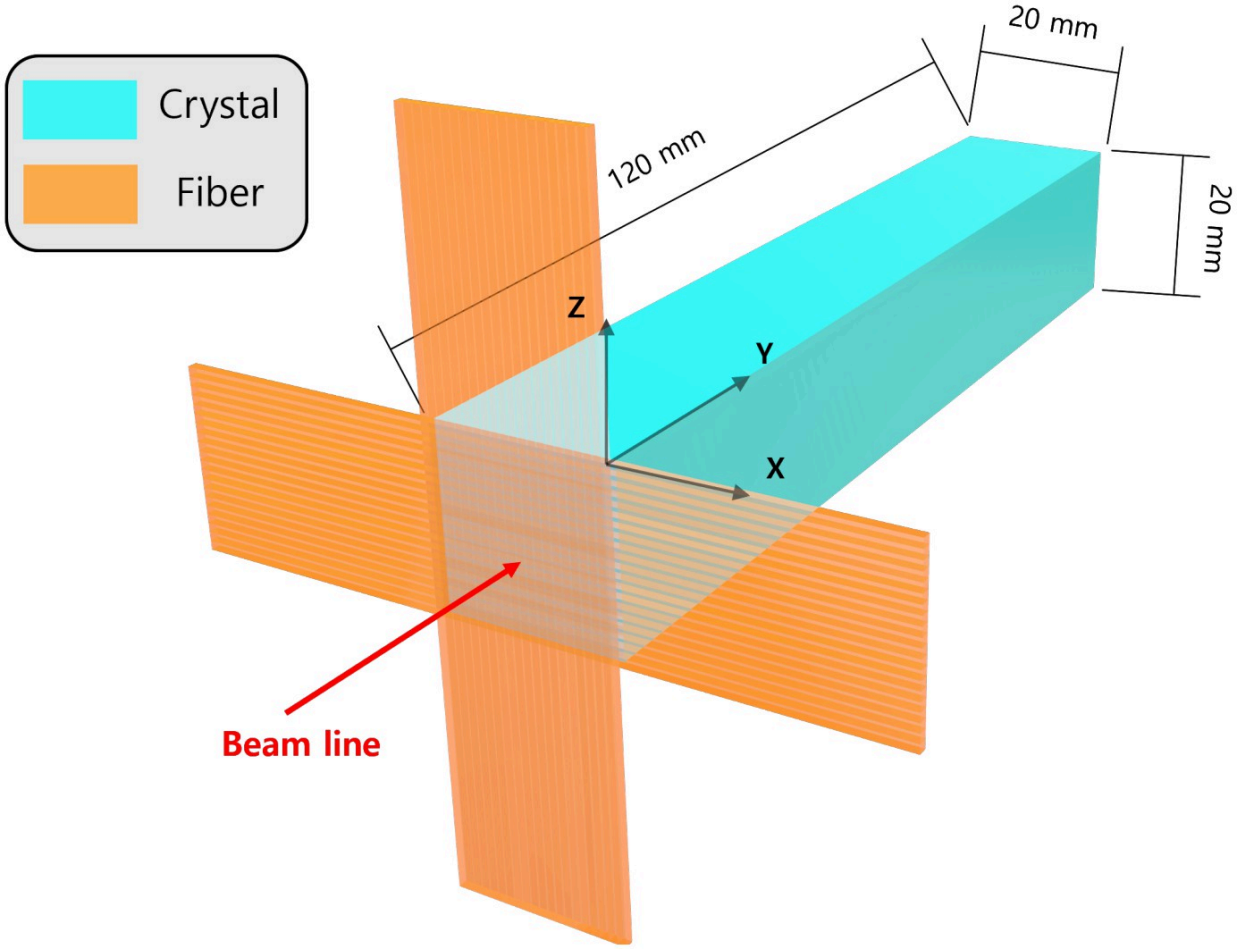
The geometry of our monitoring system consists of fiber layer and crystal. The crystal, which is sky blue part, is 20 × 20 × 120*mm*^3^. The fiber hodoscope, which is orange part, is composed by two fiber layers is place in front of crystal. The red line indicates the beam line, and the reference coordinate is located on the middle side of figure.

**Fig 4:**
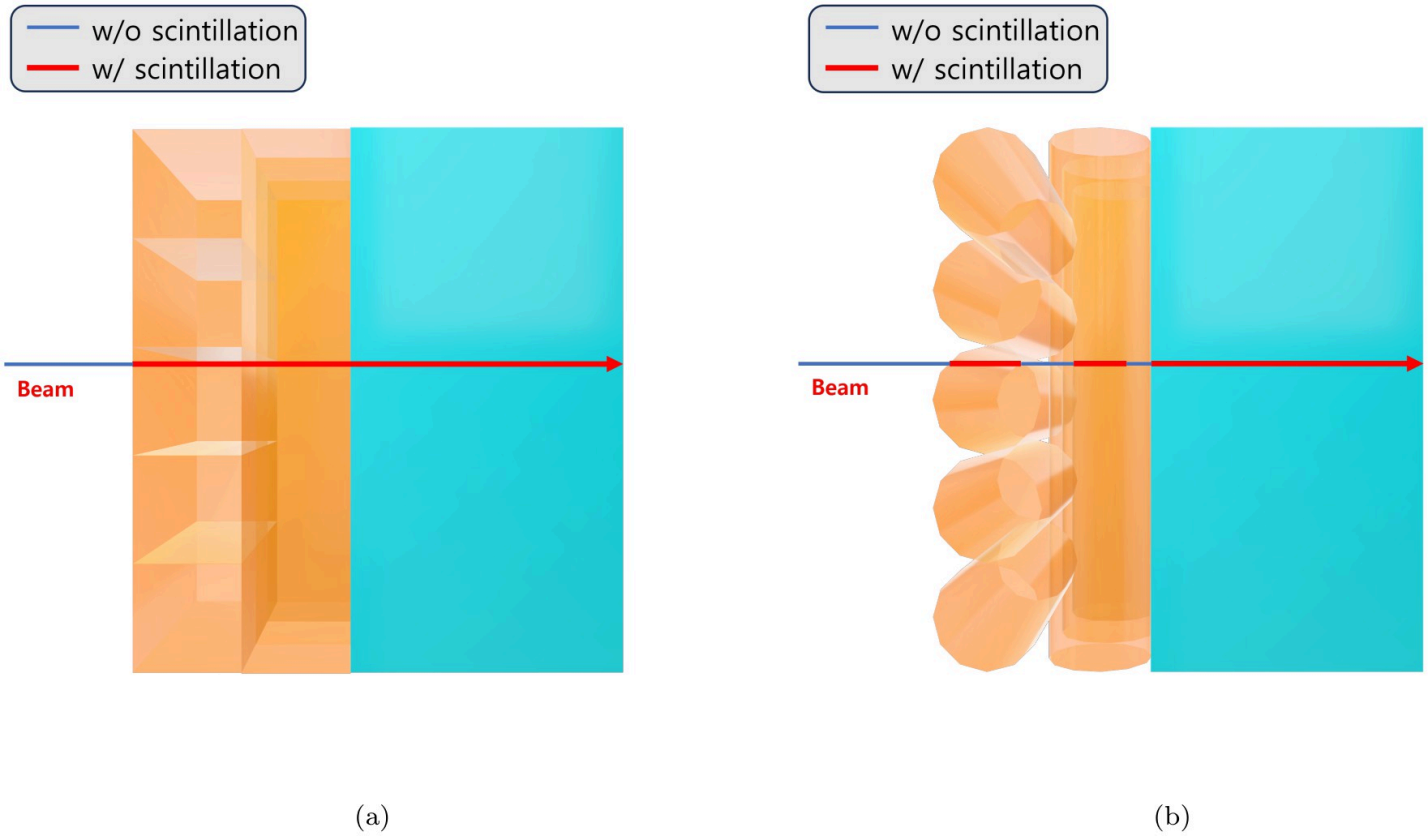
Zoom in on the boundary of the fiber layers and crystal in Fig 3. The orange part represents the fiber layer, while the sky blue part represents the crystal. The horizontal line represents the beam line, and the red line indicates the path where the beam enters the scintillation material. When the beam is on the red line, the scintillation effect occurs. (a) refers to the square type fiber, while (b) refers to the round type fiber. The round type fiber has a lot of vacancies, making it unsuitable for use as a tracker.

The fiber hodoscope consists of two scintillating fiber layers arranged vertically as shown in Fig 3. Each fiber layer reconstructs the *x*- and *z*-axes and is composed of twenty fibers and located in front of the crystal. The diameter of the used fiber (SCSF-78 from Kuraray) [35] is 1 *mm*. 2% of outermost volume is cladding (PMMA) and the remaining 98% is core (PolyStyrene). We choose the square-type fiber instead of the round-type. Since the latter has a round-shaped air volume between fibers as shown in Fig 4, the deposit energy in fiber affects this volume. So, the round-shaped air volume results in poor energy resolution. The crystals and fiber hodoscope are both scintillation materials, so when the charged particle enters the material, the photons emerge from the scintillation effect. In order to detect these photons, the Silicon Photon Multipliers (SiPM) are attached to the rear side of the crystal and to one side of each fiber in the simulation.

## Analysis

This section discusses how to reconstruct the energy and position using this beam monitoring system. With energy reconstruction, the linearity and resolution are important observables to measure the performance. These observables are calculated with all energy points of carbon and proton. In linearity, we check the response of the ratio of reconstructed and deposited energy in a crystal. When the response is consistent, this system is linear in energy reconstruction. The energy resolution indicates ability to resolve adjacent two energy peaks. The energy resolution could be represented in Eq (1) [36]. *E* and *σ* are the mean and sigma of Gaussian fitting results from energy reconstruction. And *S* is the stochastic term that is due to fluctuations of the shower from the secondary particle of the beam. The *C* is the constant term caused by the impact point of the beam.

$$\begin{array}{c} \frac{\sigma }{E}=\frac{S}{\sqrt{E}}⊕C \end{array}$$ (1)

$$\begin{array}{c} \sigma :\text{resolution}\;\text{of}\;\text{energy} \end{array}$$

$$\begin{array}{c} E:\text{reconstruct}\;\text{energy} \end{array}$$

$$\begin{array}{c} S:\text{stochastic}\;\text{term} \end{array}$$

$$\begin{array}{c} C:\text{constant}\;\text{term} \end{array}$$

Measuring the performance of position reconstruction is simple and straightforward. The reconstructed position is compared to the generated position. When the difference between both positions is within 1 *mm*^2^, we define that this system effectively reconstructs the position.

### Dataset

The dataset of this simulation includes the following contents. The beam spot is apart from 10 *cm* to the front side of the beam monitoring system. And it spreads on the *x*-*z* plane randomly within 10 × 10*mm*^2^. We count the photons which enter the SiPMs to reconstruct energy and position. To know true absorbed energy in the crystal, the position and deposited energy are used at each step of particles in simulation. We simulate carbon beam with 7 energy points between 100 to 400 MeV/u with a step size of 50 MeV/u and proton beam with 4 energy points between 50 to 200 MeV with a step size of 50 MeV. To compare position reconstruction, we also utilize the generated beam spot position. The number of generated events are summarized in Table 2. To compare the physics list, only events with QGSP_BIC are simulated using the LYSO crystal.

**Table 2:** Carbon beams are simulated with various energy; 100 to 400 MeV/u with a step size of 50 MeV/u. And proton beams also are tested; 50 to 200 MeV with a step size of 50 MeV. With FTFP_BERT physics list, 5000 events are generated for all energy points. However, only carbon beams with LYSO are simulated with QGSP_BIC physics list for comparison purpose.

	carbon (100 to 400 MeV/u) 50 MeV/u step	proton (50 to 200 MeV) 50 MeV step
FTFP_BERT	5000	5000
QGSP_BIC	5000 (only with LYSO)	-

### Energy reconstruction

**Fig 5:**
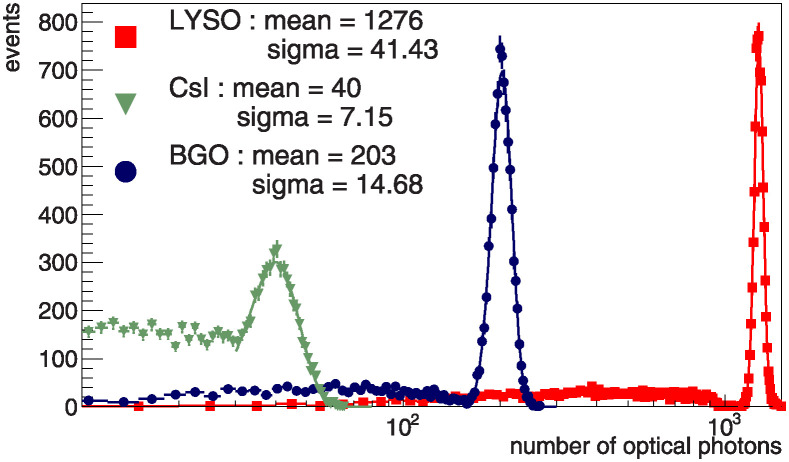
The distributions of optical photon in crystals with 0.662 MeV gamma beam are shown. The full peaks of each crystal are fitted with a Gaussian function, and their results are in top left corner of the plot. The red, green and blue lines denote LYSO, CsI and BGO respectively.

To perform energy reconstruction, the number of photons is converted to an energy dimension through a process called calibration. The 0.662 MeV gamma source from Cs-137, of which full peak clearly appears in crystal scintillator, is used in the calibration. In Fig 5, the calibration constant is calculated by dividing the energy of the gamma beam by the mean value of the full peak in the gamma spectrum, and more details are provided in Table 3. The reconstructed energy is equal to the calibration constant multiplied by the number of photons counted in the SiPM.

**Table 3:** Calibration constants determined for each crystal are shown in this table. The incident energy of gamma used in the calibration is 0.662 MeV. In gamma spectroscopy, The full peak is used in the calibration. The calibration constant is determined by dividing the incident energy by the mean value of the full peak.

	LYSO	CsI	BGO
Incident energy (MeV)	0.662		
Full peak (photons)	1276	40	202
Calibration constant (MeV/photons)	5.19 × 10^−4^	1.66 × 10^−2^	3.28 × 10^−3^

### Position reconstruction

**Fig 6:**
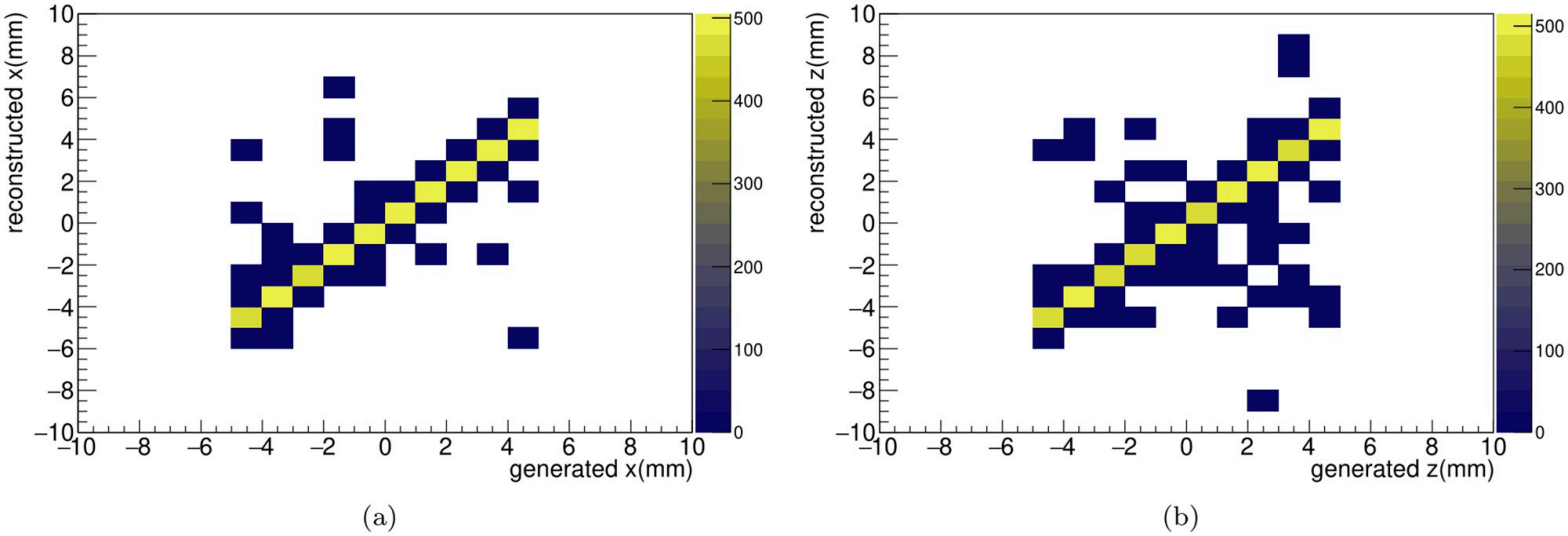
The comparison between reconstructed and generated positions in the *x*-axis (a) and *z*-axis (b) are shown. The color bar means the number of events.

For 2D position reconstruction, we design the fiber hodoscope with two layers, each dedicated to reconstructing the *x*- and *z*-axes. In each layer, the beam passes through one fiber, where the scintillation effect dominantly occurs. Thus, among the responses of fibers, only one fiber receives a significant number of photons from the scintillation. From this perspective, the position of the hot fiber indicates the measured beam position. Fig 6 diplays plots for both the *x*-axis (left) and *z*-axis (right), comparing the generation and reconstruction positions. Both positions well match within 1 *mm*. Using two layers, we reconstruct the position with two dimensional plane.

## Result and discussion

Our goal for the performance of the beam monitoring system is to achieve 1% energy and 1 *mm*^2^ position resolutions. To demonstrate the performance of the system, we measure the linearity and resolution of the reconstructed energy, and estimate how accurately the fiber hodoscope reconstructs the position of Gaussian beam spot. In addition, we discuss comparison between two physics lists.

### Energy and position reconstruction performance

**Fig 7:**
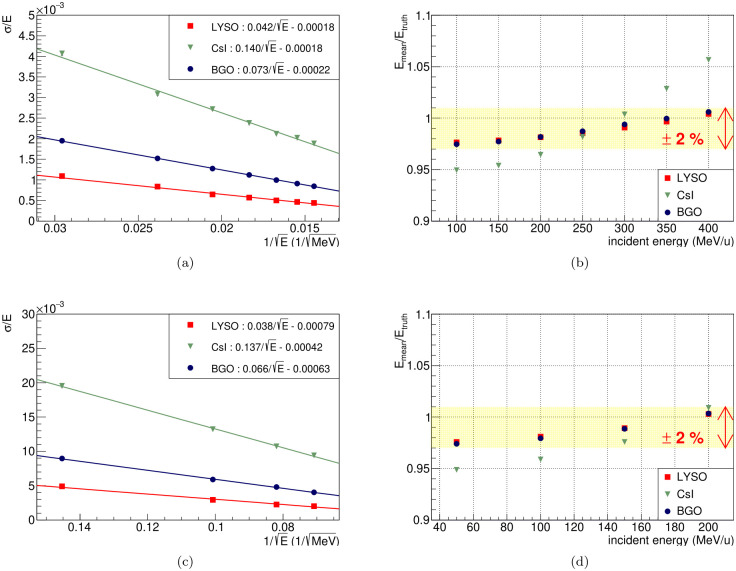
The energy resolution and linearity results are shown in the plots. The resolution plots on left side show the energy resolution by 1/$\sqrt{E}$. The linearity plots on right side indicate that the reconstructed energy is linear to the true deposited energy. Plots (a) and (b) correspond to the results for carbon beams, while plots (c) and (d) for proton beams. The red, green and blue lines denote LYSO, CsI and BGO respectively.

Fig 7 displays the results of the resolution and linearity of the carbon and proton beam. The performances of resolution determined by the Eq 1 are shown in Fig 7(a) and 7(c), which are carbon and proton results, respectively. The ratio between *σ* and mean values of reconstructed energy could be expressed as a linear function of 1/$\sqrt{E}$ of true deposited energy. The fitting results with linear function are shown in the upper right corner of each plot. Each crystal has an energy resolution of 0.061% (LYSO), 0.20% (CsI) and 0.10% (BGO) at 400 MeV/u. The performances of linearity are shown in Fig 7(b) and 7(d). The LYSO and BGO have reconstructed energies within ± 2% compared to the true deposited energies across the incident energy spectrum studied. All crystal candidates meet our goal of energy resolution.

In terms of linearity, only LYSO and BGO satisfy our criteria, while CsI does not demonstrate proper performance. All crystal candidates have a similar tendency that the responses increase at high incident energy. This is because of the Bragg peaks and the attenuation effect. The attenuation lengths of crystal candidates, which are approximately 40 *cm*, as shown in Table 1, is not negligible compared to the length of the crystal in our system. Therefore, attenuation length affects the photons generated from scintillation in the crystals. As shown in Fig 1, the depth of Bragg peak increases with higher energy beams. Therefore, the response of the high-energy beam is less affected by the attenuation effect compared to that of the low-energy beam. This results in increasing the ratio between reconstructed and true deposited energy increases at high energy beam on the plot. However, this study focused primarily on the intrinsic properties of the scintillator candidates (LYSO, CsI, and BGO) and did not explicitly consider the effects of the photo sensor and electronics.

**Fig 8:**
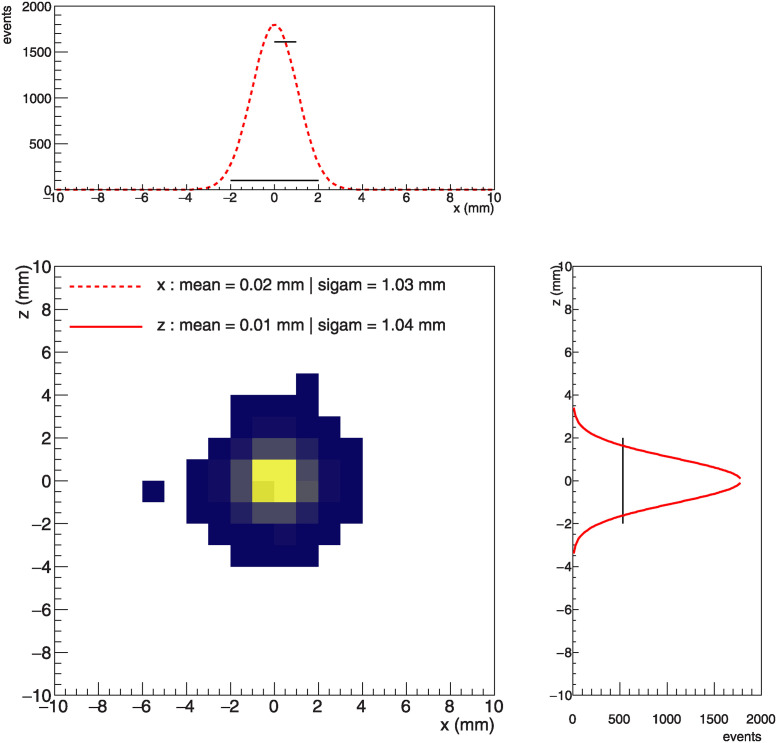
The bottom left plot shows reconstructed position using Gaussian beam spot in the *x*-*z* plane and the fitteing results in the text. The center of beam is located at (0,0) with the yellow region highlighting a higer beam intensity compared to the dark plue regions. The top and right plots represent the projection onto *x*-axis and *z*-axis, respectively, fitted with a Gaussian function. The *σ*s of the incident beam are set to 1 *mm*, and those of reconstructed position are within 5% in comparison with the incident beam.

To evaluate the performance of position reconstruction, we generate a beam using a Gaussian distribution, with a mean of 0 and a sigma of 1 *mm* along the *x*-axis and *z*-axis. The reconstructed position for each axis is then fitted with a Gaussian distribution, as illustrated in Fig 8. The mean and *σ* values of Gaussian fitting with reconstructed position of both axes are shown in the bottom left plot of Fig 8. The errors of the reconstructed position are within 5% of the incident values, indicating that the performance of the fiber hodoscope meets our goal.

### Comparison of the physics lists

**Fig 9:**
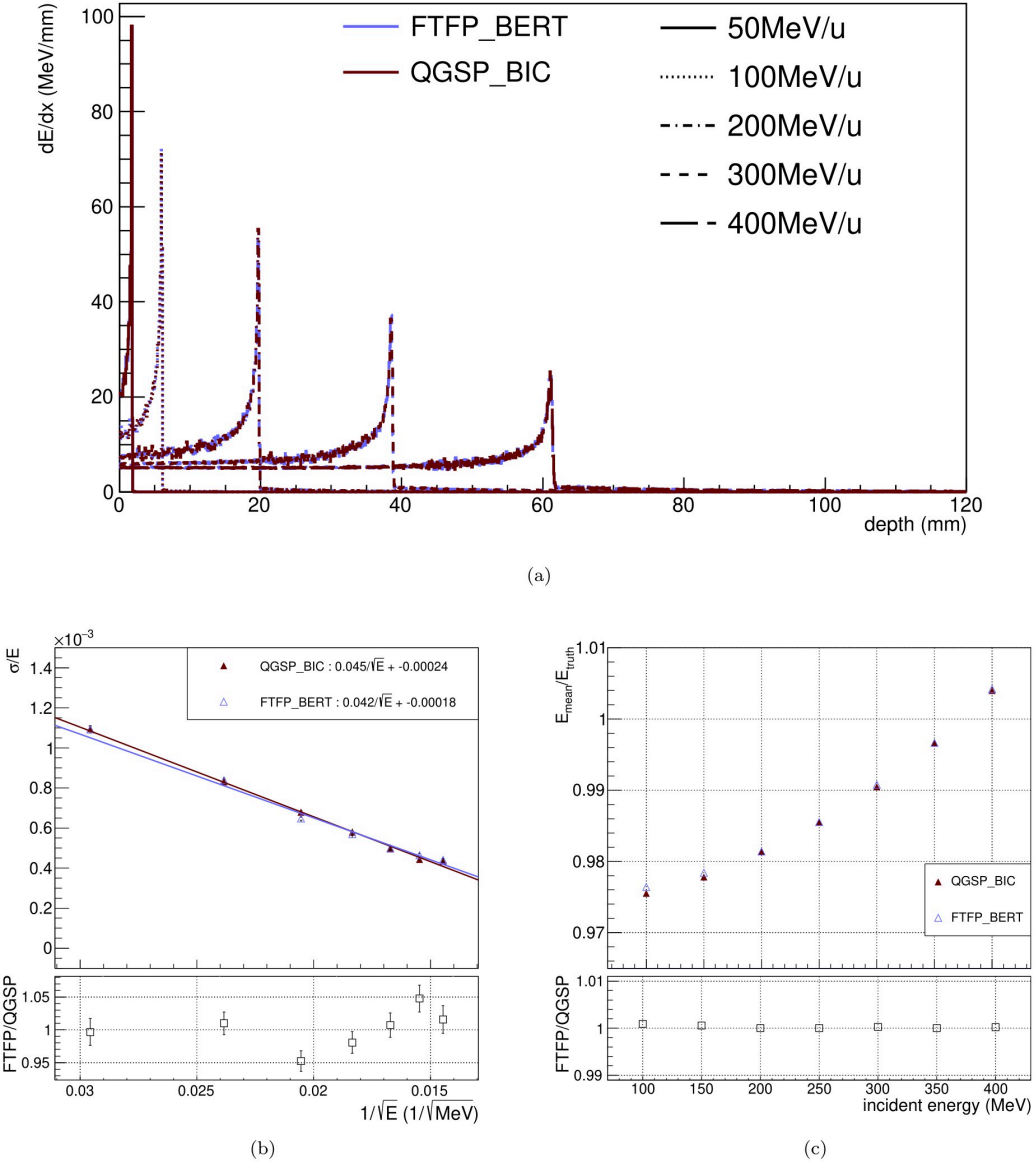
(a) This figure shows the comparison of the Bragg peaks between FTFP_BERT and QGSP_BIC. The blue and red lines denote FTFP_BERT and QGSP_BIC, respectively. The incident beam energies are distinguished by style of lines. From front side of crystal of beam direction to Bragg peak, the difference of both lists is within ± 5%. The energy resolution and linearity are also compared between FTFP_BERT and QGSP_BIC in (b) and (c), respectively. Both are in good agreement within ± 5%.

We compare the FTFP_BERT and QGSP_BIC physics lists with LYSO, and the results are shown in Fig 9. As shown in Fig 9(a), we compare the Bragg peak between the two physics lists, and both physics lists are in good agreement within ± 5%. The main difference in physics lists lies in hadron physics. In contrast to FTFP_BERT, the QGSP_BIC takes into account for the color flow in QCD and light ion reaction [37, 38]. However, since the Bragg peak is produced by the non-relativistic Bloch Bethe Eq (2) [39], there is no significant difference in Bragg peak between two physics lists. The performances of energy linearity and resolution are also compared, as shown in Fig 9(b) and 9(c). The ratio between physics lists in both results is within ± 5%. Therefore, we conclude that the difference between two physics lists is negligible in this study.

$$\begin{array}{c} -\frac{dE}{dx}=\frac{4\pi }{m_{e}c^{2}}\frac{nZz^{2}}{\beta^{2}}{\left(\frac{e^{2}}{4\pi \epsilon_{0}}\right)}^{2}\left[ln\left(\frac{2m_{e}c^{2}\beta^{2}}{I}\right)\right] \end{array}$$ (2)

$$\begin{array}{c} \frac{dE}{dx}:\text{stopping}\;\text{power} \end{array}$$

$$\begin{array}{c} \epsilon_{0}:\text{permittivity} \end{array}$$

$$\begin{array}{c} Z:\text{material}\;\text{atomic}\;\text{number} \end{array}$$

$$\begin{array}{c} n:\text{Avogadro}\;\text{constant} \end{array}$$

$$\begin{array}{c} z:\text{beam}\;\text{atomic}\;\text{number} \end{array}$$

$$\begin{array}{c} v:\text{speed}\;\text{of}\;\text{the}\;\text{particle} \end{array}$$

$$\begin{array}{c} c:\text{speed}\;\text{of}\;\text{light} \end{array}$$

$$\begin{array}{c} \beta :\text{ratio}\;v\;\text{to}\;c\;\text{of}\;\text{beam} \end{array}$$

$$\begin{array}{c} I:\text{mean}\;\text{excitation}\;\text{energy} \end{array}$$

$$\begin{array}{c} m_{e}:\text{electron}\;\text{mass} \end{array}$$

## Conclusion

Compared to photon treatment, CIRT has significant advantages as it precisely targets tumors while minimizing collateral damage to nearby healthy tissues and organs. To guarantee correct delivery of CIRT, cutting-edge technologies for precise energy measurement and real-time monitoring are essential. We aim for the 1% energy and 1 *mm*^2^ position resolutions of the beam monitoring system in order to measure the beam performance of CIRT. We propose the system composed of a scintillation crystal and fiber hodoscope. The candidates for the scintillation crystal are LYSO, CsI, and BGO. The size of crystal, which is 20 × 20 × 120*mm*^3^, is determined with consideration of energy deposition. The fiber hodoscope consists of 1 *mm* thickness scintillation square type fibers and the fibers are placed vertically to facilitate 2D reconstruction. We validate our beam monitoring system’s performance using GEANT4 simulation. For estimating performance of energy reconstruction, we measure the linearity and resolution. All crystal candidates are achieved our goal of the energy resolution. The energy resolution of each crystal with 400 MeV/u carbon beam is 0.061% (LYSO), 0.20% (CsI) and 0.10% (BGO). The LYSO and BGO are linear within ± 2%. Our goal for position resolution is satisfied with the fiber hodoscope design. Using a two-dimensional Gaussian beam spot, the reconstructed position has an uncertainty within 5% compared to the generated position.

**Fig 10:**
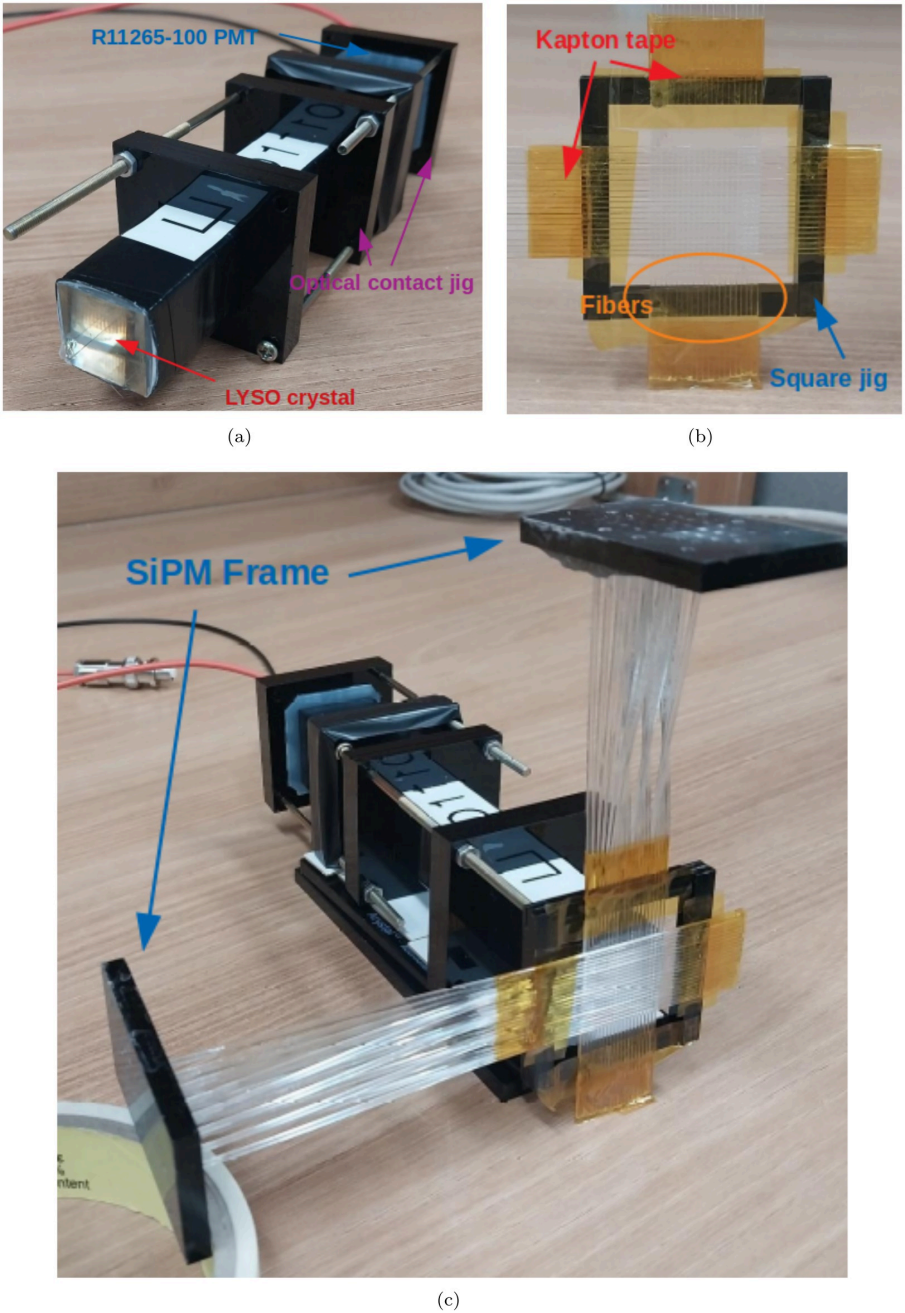
The prototype beam moniotring system based on geometry shown in Fig 3 is built. (a) The crystal (LYSO) and PMT (R11265-100) are assembled using black square jigs. (b) The fiber hodoscope is composed of two fiber layers and jig for arranging the layers and a jig. (c) The prototype with fiber hodoscope and crystal are shown.

In this study, the effect of photo-sensor or electronics is not considered. So, we produce the prototype detector. Based on designed geometry, a newly built prototype system in Fig 10 is described as follows. First, in Fig 10(a) the black cuboid with frames is shown. It is LYSO (from Epic-Crystal) wrapped with teflon tape for reflection (black tape: insulating tape). We use the photomultiplier tube (PMT; R11265-100 from Hamamatsu) [40] as the readout to measure the electronic signal produced by multiplying the scintillation photons. The jigs are utilized to optimize optical contact between crystal and PMT.

Second, the fiber hodoscope is shown in Fig 10(b). Each fiber is aligned in parallel and the twenty fibers are wrapped with yellow Kapton tape. The SiPM (S14160-1310PS from Hamamatsu) [40], which is 1.3 × 1.3*mm*^2^, is used as readouts of fibers. To achieve high granularity, we select SiPM which has small dimensions. To improve optical contact between the fiber and the SiPM, we manufacture a black frame for the fiber and a customized PCB board for the SiPM. The fibers are fixed with the optical cement (EJ-500 from Eljen Technology) [42] in the frame rather than using other epoxy, because the optical cement has a similar refractive index to that of the fiber and glass in SiPM. We make a square jig for arranging two layers vertically. Finally, the prototype is assembled with the fiber hodoscope and crystal, as shown in Fig 10(c).

## Supporting information

S1 Data (Zip)
